# Novel Technique for Small Bone Fixation Using Kirschner Wire Reduction and Cortical Screws

**DOI:** 10.7759/cureus.97052

**Published:** 2025-11-17

**Authors:** Oluwatoba J Akinleye, Jared Sasaki, Gabriela Rodriguez, Logan W Carr

**Affiliations:** 1 Orthopaedic Surgery, New York Medical College, Valhalla, USA; 2 Orthopaedics, New York Medical College, Valhalla, USA; 3 Plastic and Reconstructive Surgery, Conemaugh Memorial Medical Center, Johnstown, USA

**Keywords:** bone tunnel technique, kirschner wire (k-wire), screw fixation, small bone fractures, vascular injury

## Abstract

Vascular injury is a common complication in the reduction of small bone fractures of the hand. Proper technique and management during fracture fixation can help mitigate this risk. We report a case that discusses the use of a Kirschner wire (K-wire) technique for both initial fracture reduction and subsequent screw fixation in small bones, utilizing a bone tunnel created by the K-wire. This approach offers a preventative strategy to reduce the risk of vascular compromise, minimize bone tissue loss, and improve the accuracy of screw placement.

## Introduction

Effective reduction of small bone fractures has been demonstrated using a variety of techniques, such as fixation with Kirschner wires (K-wires), screws alone or in combination with plates [1-4], and external fixation [5]. Conventional management involving K-wires primarily limits its application to initial reduction and fixation, followed by definitive fixation with screws. While K-wires alone do not provide compression across fracture segments, they have been shown to be effective in definitive fixation [6]. Screw and plate fixation offers superior stabilization, but in situations where space is limited, screws alone are preferred. Plates require larger incisions and increase soft tissue disruption, making them less ideal in such cases. Small bone fractures of the hand are among the most common upper extremity injuries, and achieving stable fixation in such confined spaces remains a frequent technical challenge for surgeons [6].

Achieving stable fixation with screws typically involves drilling a hole parallel to the temporarily placed K-wires, guided by a drill sleeve. Following screw placement and fluoroscopic confirmation, the K-wires are safely removed. In cases where the bony fragment is very small, there is minimal surface area to accommodate both the insertion of temporary K-wires and a separate hole for screw placement. Furthermore, drilling additional holes increases the risk of vascular compromise or fracture of the small bone fragment.

Here, we describe a novel technique that uses the K-wires to guide the insertion of a screw. This allows avoidance of additional drilling by utilizing the hole created by the K-wire. Additionally, this technique minimizes the risk of losing visualization of a drilled hole by soft tissue concealment.

## Case presentation

**Figure 1:**
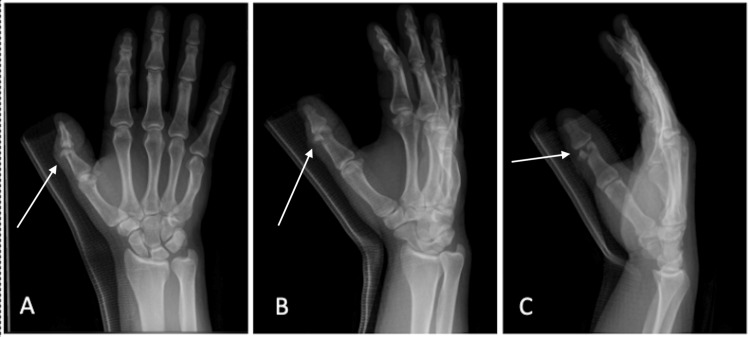
Anteroposterior (AP) (A), oblique (B), and lateral (C) views of the right hand demonstrating a fracture at the head of the proximal phalanx of the thumb (arrows).

The patient presented following an accident involving a circular saw that resulted in an open fracture of the right thumb. The laceration extended radially from the dorsal aspect of the interphalangeal (IP) joint to the volar surface. The patient exhibited decreased sensation of the radial aspect of the thumb and limited active extension at the IP joint, while flexion remained normal at the IP and metacarpophalangeal (MCP) joints. Capillary refill was under two seconds, with no evidence of vascular compromise. X-rays revealed a unicondylar, comminuted intra-articular proximal phalanx fracture on the radial side of the digit at the IP joint, consistent with a type II phalanx fracture (Figure 1). Of note, the bony injury’s angulation on X-ray was similar to that of the laceration, and there was a comminuted divot of bone loss from the saw blade proximal to the condylar fragment.

The laceration was extended to allow sufficient exposure of the injured structures. After thorough irrigation, reduction of the fracture was achieved. Due to the size of the fragment, 0.035-inch (0.889 mm) K-wires were selected for both preliminary and definitive fixation using 1.3 mm Synthes screws (DePuy Synthes, USA) (Table 1). Since cannulated screws are not available in this size, the K-wire path was used as a guide for screw insertion.

**Table 1:** K-wire sizing chart demonstrating which K-wire diameters can be used as a tunneling substitute for the drill bit typically used to create the screw track. K-wire sizes are conventionally reported in inches, with the corresponding millimeter values provided here for comparison. For instance, the 0.035-inch (0.889 mm) K-wire used during fixation created a tunnel comparable in size to the 1.0 mm drill bit normally used for placement of the 1.3 mm Synthes screw.

K-wire		Synthes	
K-wire size (inches)	Conversion to mm	Corresponding screw size diameter (mm)	Usual drill bit diameter (mm)
0.035	0.889	1.3	1
0.045	1.143	1.5	1.1
0.054	1.3716	Not available	Not available
0.062	1.5748	2.0	1.5

**Figure 2:**
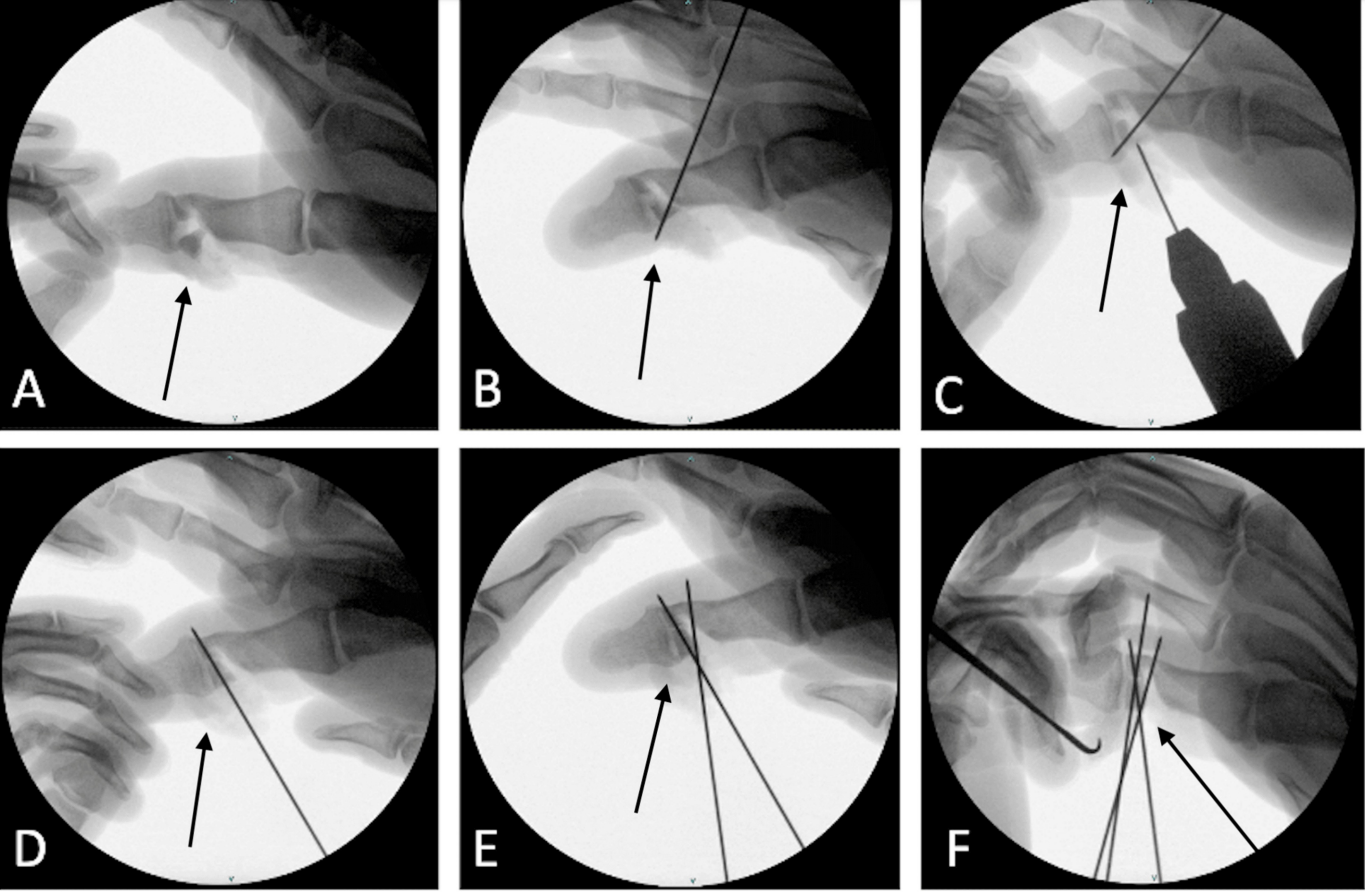
Intraoperative fluoroscopic images demonstrating open preliminary fixation. (A) Identification of the fracture fragments after irrigation and debridement (arrow). (B) Placement of a K-wire to capture the fragment (arrow). (C-D) Positioning and placement of a transverse K-wire, followed by removal of the preliminary K-wire (arrow). (E-F) Placement of the second and third transverse K-wires to complete the preliminary fixation (arrow).

A single K-wire was placed at an oblique angle from the ulnar to the radial side of the thumb to capture the fragment prior to inserting the transverse wires, which were later replaced by screws. Then two K-wires were placed transversely from the radial to ulnar side across the distal aspect of the proximal phalanx of the thumb. These wires were used to pin the fractured radial condyle of the phalanx to the ulnar condyle, which remained intact with the proximal phalanx. Reduction was confirmed using orthogonal X-ray images (Figure 2).

**Figure 3:**
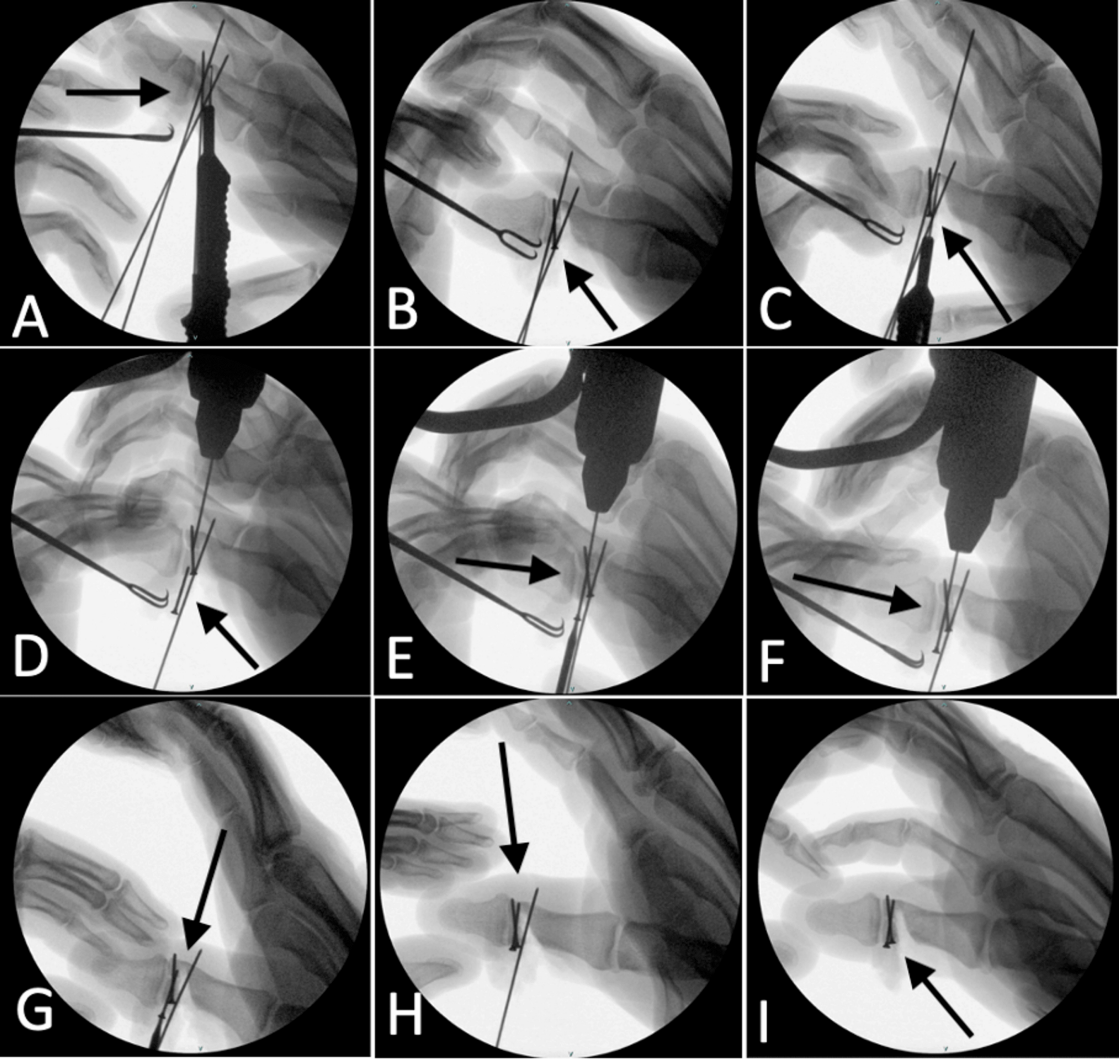
Intraoperative fluoroscopic images demonstrating definitive fixation of the fracture fragment. (A) Depth-gauge measurement for the first screw (arrow). (B) Placement of the first screw through the K-wire tunnel (arrow). (C) Depth-gauge measurement for the second screw (arrow). (D-F) Gradual withdrawal of the K-wire in the ulnar direction with simultaneous advancement of the screw within the K-wire tunnel (arrow). (G-I) Final stages of placement of the second screw (arrow).

A depth gauge was used in combination with fluoroscopic image guidance to select the appropriate length screw (Figure 3A). Once the appropriate 1.3 mm Synthes cortical screw was selected, we focused on obtaining the definitive fixation via the K-wire tunnel (Figure 3). This was achieved by initially withdrawing the K-wire from the contralateral side, leaving a minimal segment on the radial side protruding from the bone to avoid loss of visualization. Next, the tip of the cortical screw was placed over the tip of the K-wire and approximated to follow the tunnel created by the K-wire. The K-wire was gradually withdrawn from the opposite side while the screw was simultaneously inserted and rotated into the tunnel created by the K-wire (Figures 3D-3F). The technique was repeated for the second K-wire (Figures 3G-3I).

**Figure 4:**
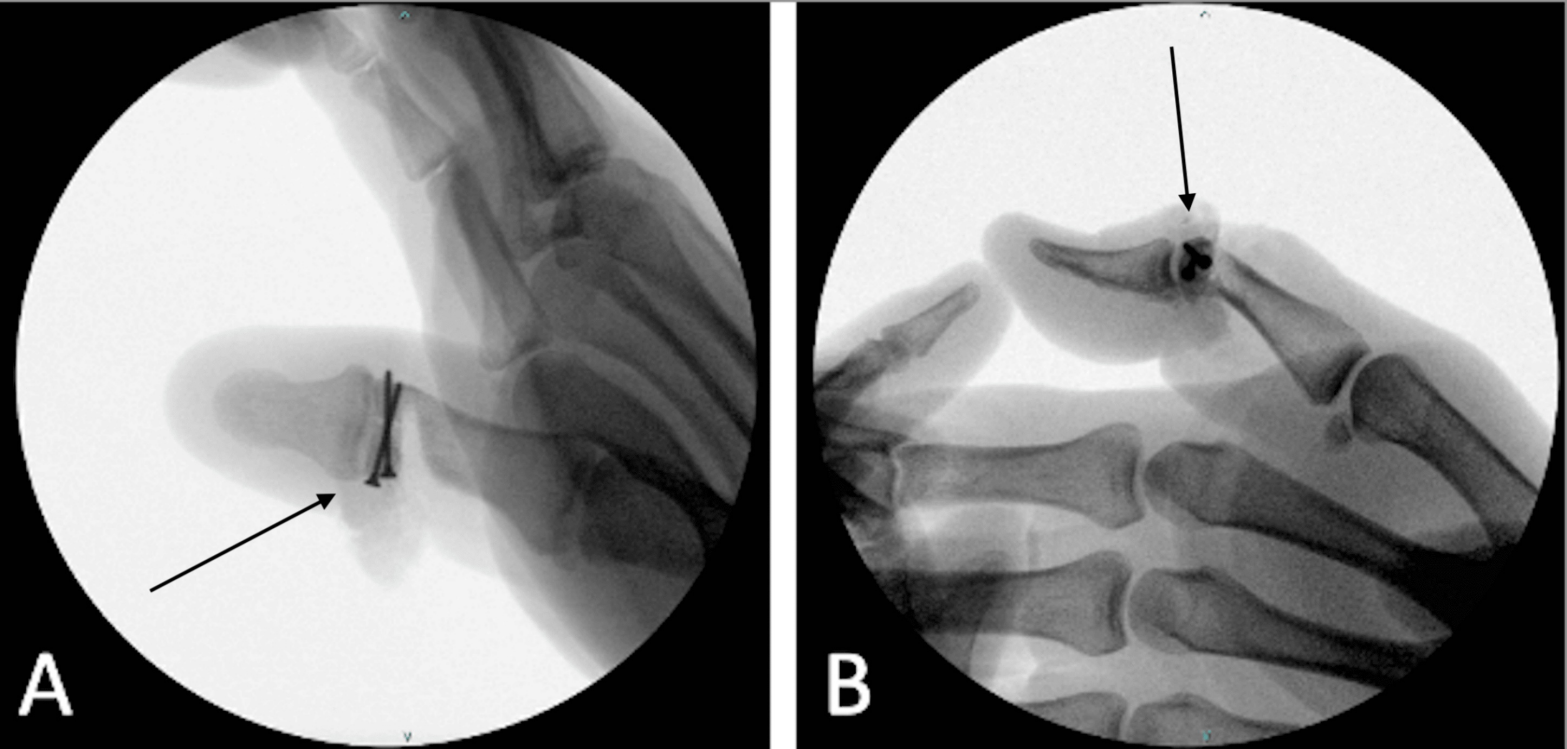
Final intraoperative fluoroscopic images demonstrating definitive fixation of the fracture (arrows). (A) Anteroposterior view. (B) Lateral view.

Definitive fixation was confirmed via intraoperative fluoroscopic images (Figure 4). The divot left from the saw blade measured approximately 0.25 inches and was filled with bone matrix to encourage healing. Subsequent repair of partial flexor pollicis longus (FPL) and complete extensor pollicis longus (EPL) tendon lacerations, as well as nerve coaptation, were performed prior to closure with suture.

## Discussion

Phalanx fracture fixation is traditionally performed using K-wire stabilization followed by screw fixation or the use of cannulated screws [7-9]. However, due to the small nature of the anatomy and the fine precision required for stabilization with the wires and screws used, traditional methods have inherent limitations. In this case report, we present a novel approach designed to mitigate these challenges. By utilizing the K-wire tunnel as the primary entry pathway for the screw, the need for additional drilling is minimized, thereby reducing bone loss and lowering the risk of fracture, vascular injury, or joint destabilization from mechanical or thermal complications related to K-wire use [10-14].

The technique described here shares similarities with the use of cannulated screws, commonly applied in orthopedic surgeries involving larger drill bits. In these procedures, a K-wire is placed, and a cannulated screw is placed over the K-wire. The K-wire acts as a guide and is removed following screw fixation. While commercially available cannulated screws start at a minimum diameter of 2 mm, our technique offers a practical alternative for scenarios requiring screws smaller than 2 mm.

A significant challenge associated with the small wires and screws traditionally used in this context is the potential loss of visualization after drilling, which can lead to additional holes that destabilize the joint. Our approach addresses this by allowing the surgeon to use the existing K-wire tunnel as a guide for screw placement, avoiding the need for a separate drilled hole. If the tunnel is temporarily obscured, the K-wire can be advanced to regain the entry point before proceeding with screw insertion.

Furthermore, in cases where fragment surface area is insufficient for traditional fixation methods, our technique provides a viable alternative [15,16]. This approach is adaptable to other procedures requiring small fragment fixation, such as in pilon fractures of the finger. In this procedure, hemi-hamate arthroplasty requires the removal of the comminuted fragments of the PIP joint with subsequent fixation of a graft donated from the hamate bone, which must be fixed with small-caliber screws. Due to the small size of the graft and the nature of the procedure, minimizing damage and excess holes in the hamate graft is crucial. Using our method of screw placement within the K-wire tunnel could preserve more of the graft, potentially enhancing graft survival and reducing fracture risk.

This technique could inspire future innovations in both K-wire and screw technology, specifically for procedures requiring sub-2 mm screws. For instance, screws too small to be cannulated could be developed with a male-female interface, allowing seamless integration with the distal end of a K-wire. Such advancements would make K-wire-guided screw placement more efficient and versatile, expanding the range of minimally invasive fixation options available for small bone fractures. Although follow-up data for this case were not available, such information would be valuable to assess long-term functional outcomes, fixation stability, and the reproducibility of this technique in future reports.

## Conclusions

In summary, we present a novel technique for screw placement in open reduction and internal fixation (ORIF) procedures. The method entails placing the screw through the exit point of the K-wire as the surgeon simultaneously withdraws the wire. This approach offers several advantages, including reduced damage to the joint, decreased risk of vascular injury, and improved visualization of the small holes during surgery. This technique, whether performed in practice, has not yet been described in the literature and will add to the existing body of work. Future reports, including postoperative follow-up, will be important to further validate and assess long-term outcomes of this technique.
